# Vaccination Attitudes Examination (VAX) Scale: a Bifactor-ESEM approach in a youth sample (15–24 years)

**DOI:** 10.1186/s40359-023-01388-9

**Published:** 2023-10-23

**Authors:** Veljko Jovanović, Milica Lazić

**Affiliations:** Department of Psychology, Faculty of Philosophy, University of Novi Sad, Dr Zorana Đinđića 2, Novi Sad, Novi Sad, 21 000 Serbia

**Keywords:** Vaccination attitudes, Vaccination Attitudes Examination (VAX) Scale, Bifactor-exploratory structural equation modeling, Vaccine hesitancy, Vaccination behavior

## Abstract

**Background:**

The Vaccination Attitudes Examination (VAX) Scale is a widely used scale designed to measure general attitudes toward vaccinations. However, evidence for the VAX’s structural, convergent, and discriminant validity is still limited, especially in youth samples.

**Methods:**

The present study examined the psychometric multidimensionality and evidence of convergent and discriminant validity of the VAX using the bifactor-exploratory structural equation modeling approach (bifactor-ESEM). Using a sample of 803 Serbian adolescents and young adults (*M*_age_ = 18.23, *SD*_age_ = 2.66, age range = 15–24 years, 59.2% female), we contrasted the original four-factor model of the VAX with alternative solutions (ESEM, bifactor-CFA, and bifactor-ESEM), and investigated associations between vaccination attitudes and a variety of external criteria.

**Results:**

The results supported the bifactor-ESEM solution with one general factor of vaccination attitudes and four specific factors (Mistrust of vaccine benefit, Worries about unforeseen future effects, Concerns about commercial profiteering, and Preference for natural immunity) as the best representation of the data. The general factor was well-defined, and three specific factors showed good validity and specificity after the general factor was taken into account. The results of convergent validity analyses showed that the general factor of vaccine attitudes and one specific factor (Mistrust of vaccine benefit) were good predictors of vaccine conspiracy beliefs, attitudes towards COVID-19 vaccination, intention to get vaccinated against COVID-19, and trust in healthcare. The remaining three specific factors’ contributions to external criteria were generally weak and nonsignificant. Evidence of the discriminant validity of the VAX scores was supported by weak positive associations of the general factor with medical fears and paranoid worry.

**Conclusions:**

The present findings indicate that distinguishing general and specific components of vaccination attitudes offers a more nuanced assessment and understanding of vaccination attitudes.

**Supplementary Information:**

The online version contains supplementary material available at 10.1186/s40359-023-01388-9.

## Background

Understanding predictors of vaccine hesitancy has become one of the key challenges in the field of public health in recent years, as an increasing number of people are becoming skeptical toward vaccines [1], even though the effects of vaccine refusal or a delay in receiving vaccines can have severe consequences and detrimental effects on health [2]. According to the World Health Organization [3], regular vaccination programs have been severely disrupted over the last three years; global vaccination coverage has dropped from 86% in 2019 to 81% in 2021, and it is estimated that 25 million children under the age of one have not received basic vaccines. If vaccination rates are reduced, even for short periods during crises, there may be more frequent outbreaks of vaccine-preventable diseases such as polio or measles, which had almost been eradicated [4]. These issues have placed a high priority on research into the complex nature of vaccine hesitancy determinants, including attitudes toward vaccination [5], conspiracy beliefs [6], generalized and particularized trust [7], and vaccine-related knowledge [8], to name a few.

Recent studies suggest that vaccine hesitancy is widespread, with various reasons for opposing vaccination acceptance [9]. Given that a deeper understanding of the causes underlying vaccine hesitancy almost inevitably involves examining attitudes towards vaccination [10], the development of valid and reliable questionnaires aimed at measuring vaccination attitudes has become a critical task for researchers in recent years. To date, a variety of measures have been developed to assess attitudes toward vaccines against specific infectious diseases such as HIV (e.g., HIV Vaccine Attitudes Scale) [11], human papillomavirus (e.g., HPV Attitude Questionnaire) [12], and COVID-19 (e.g., COVID-Vaccination Attitude Scale) [13]. Most scales developed to assess vaccination attitudes are uni- or two-dimensional, measure overall favorable and unfavorable attitudes about immunization against certain diseases [14], and are mostly designed for parents (e.g., Carolina HPV Immunization Attitudes and Beliefs Scale) [15]. This lack of multidimensional tools for assessing general attitudes about vaccines among adults led Martin and Petrie [16] to develop the Vaccination Attitudes Examination (VAX) Scale, a 12-item measure of attitudes towards vaccines and immunization. The aim of the VAX was to provide researchers with a brief, multifaceted questionnaire for measuring general attitudes towards vaccination. This in turn, might help them understand the attitudes underlying vaccination hesitancy and provide information for those designing public health campaigns to increase vaccination rates. Martin and Petrie relied on the results of previous research, which showed low vaccination rates might reflect general skepticism about medical interventions, as well as specific worries about vaccine safety, mistrust of vaccine benefit, concerns about commercial profiteering, and the motivations of those who advocate for vaccination [17, 18].

The results of two initial validation studies [16] provided support for the four-factor oblique structure of the VAX, with four strongly correlated factors labeled *Trust/Mistrust of Vaccine Benefit*, *Worries about Unforeseen Future Effects*, *Concerns about Commercial Profiteering*, and *Preference for Natural Immunity*. Research pre-dating COVID-19 rarely made use of the VAX, but this changed dramatically during the ongoing pandemic. For example, based on a citations count retrieved from SCOPUS on August 25, 2023, the original paper describing the development of the scale received 10 citations during 2017–2020, 36 citations in 2021, and 85 citations in 2022.

However, despite the widespread use of the VAX, evidence for its structural, convergent, and discriminant validity is still limited. To date, the scale has been translated into several languages, and validation studies have been published using samples from Spain [19, 20], Italy [21, 22], Colombia [23], Turkey [24], Romania [25], the UK [26], South Korea [27], and France [28]. All these studies applied a confirmatory factor analysis (CFA) to test the factor structure of the VAX and reported support for the scale’s original four-factor structure. In most studies, only the original four-factor model was tested [20–22, 24–27], whereas Espejo et al. [19, 23] and Eisenblaetter et al. [28] also tested the single-factor model. However, to our knowledge, no study to date has investigated whether vaccination attitudes assessed by the VAX should be conceptualized as a general factor underlying mistrust of vaccine benefit, worries about unforeseen future effects, concerns about commercial profiteering, and preference for natural immunity, or if these four aspects of vaccination attitudes are distinct and relatively independent dimensions. This question is not well addressed by a traditional CFA for several reasons. As argued by Marsh and colleagues [29], fixing cross-loadings to zero in CFA (i.e., in the commonly tested four-factor CFA model of the VAX structure) may be too restrictive for multidimensional measures, such as the VAX. It can be expected that VAX items designed to measure each of the four partially overlapping domains of vaccination attitudes are also associated with other not-target but conceptually related domains. Therefore, applying models that allow free estimation of all cross-loadings might lead to a more meaningful representation of the VAX structure. Additionally, the latent factor correlations are typically inflated in the CFA models without cross-loadings allowed [30]. The results of previous studies using the VAX regarding latent factor correlations suggest that some factors are poorly discriminated and that this may involve multicollinearity in the estimation of associations with other variables. For example, the range of latent factor correlations in an Italian sample was 0.514–0.812 [21], in the French sample between 0.62 and 0.78 [28], and correlations above 0.65 between some latent factors were observed in other studies [19, 23]. The original development study [16] reported latent factor correlations ranging from 0.65 to 0.75. Such strong correlations suggest the existence of a general overarching construct of vaccination attitudes, but to our knowledge, this assumption has not been tested to date. Furthermore, the four VAX subscales have typically yielded highly similar correlations with the convergent measures in previous studies [21, 28], suggesting that these four dimensions of vaccination attitudes might lack specificity over and above the general factor of attitudes toward vaccination. The present study addressed the limitations of previous structural validity studies of the VAX by applying the bifactor model, exploratory structural modeling (ESEM), and the bifactor-ESEM approaches [31]. We followed Morin et al.’s [32] recommendations to evaluate the presence of two types of construct-relevant psychometric multidimensionality, with one related to the presence of conceptually related constructs and the other to the presence of hierarchically ordered constructs.

Another major shortcoming of previous studies on the VAX is limited evidence for the convergent and discriminant validity of the scale scores. The convergent validity of the scale has been evaluated only in a few studies. For example, Eisenblaetter et al. [28] found that VAX subscale scores had strong correlations with vaccine conspiracy beliefs and parent attitudes about childhood vaccines, and moderate to strong correlations with various beliefs about medicines. In addition, two studies [19, 23] examined the associations between VAX scores and the vaccination status assessed by the question “Have you been vaccinated against COVID-19?” with “Yes” and “No” response options, while Bruno et al. [21] and Huza [25] used mostly single-item measures for convergent validity analyses. Finally, some studies [20, 26, 27] used the total VAX score in the convergent validity analyses, although the four-factor structure of the scale was supported and there was no evidence of the scale’s unidimensionality. To date, only a limited number of studies have evaluated the psychometric properties of the VAX in youth samples [23, 33]. Furthermore, the vast majority of questionnaires designed to measure vaccine-related constructs have rarely been validated in samples comprising adolescents and young adults. This is surprising given that understanding vaccine willingness and hesitancy determinants (such as attitudes towards vaccines and immunization) among adolescent and young adult remains a serious public health challenge, due to low vaccine uptake compared to children [34]. Vaccine hesitancy in these age groups is complex and shaped by numerous factors [35], which highlights the importance of having psychometrically sound instruments for assessing factors influencing vaccine hesitancy.

## The Present Study

Given the incomplete evidence for the structural, convergent, and discriminant validity of the VAX in previous studies and limited evidence on the scale’s psychometric performance in youth samples, the main goals of the present study were twofold: First, we wanted to investigate the factor structure of the Serbian translation of the VAX in a youth (15–24-year-olds) sample. In line with previous studies, we expected to find support for the original four-factor structure of the VAX with four correlated factors. However, this scale consists of four theoretically closely associated dimensions of vaccination attitudes, so we also expected bifactor and ESEM models to provide a good fit to the data. Second, we aimed to evaluate evidence on the convergent and discriminant validity of the VAX scores in relation to measures of intention to get vaccinated against COVID-19, attitudes towards COVID-19 vaccination, vaccine conspiracy beliefs, trust in healthcare, medical fears, and paranoid worry. Evidence for the convergent validity of the VAX scores would be supported by moderate to strong negative associations with intention to get vaccinated against COVID-19 [5], attitudes towards COVID-19 vaccination [36], trust in healthcare [37], and moderate to strong positive associations with vaccine conspiracy beliefs [28]. Based on the findings of previous studies, we expected that *Mistrust of Vaccine Benefit* and *Worries about Unforeseen Future Effects* would be the strongest correlates of unwillingness to vaccinate against COVID-19 [5], and that *Concerns about Commercial Profiteering* would be the strongest correlate of vaccines conspiracy beliefs [28]. Evidence of discriminant validity would be indicated by weak or nonsignificant associations with medical fears and paranoid worry—two constructs that were expected to show weak associations with vaccination attitudes [38, 39].

## Methods

### Sample and Procedure

The sample included 803 Serbian youths (mean age = 18.23, *SD* = 2.66, age range = 15–24 years, 59.2% female). A more detailed description of the study sample is shown in Additional File 1. Participants were recruited from July to October 2022 and asked to participate in a project on vaccine hesitancy among young people in Serbia. The questionnaires were administered either online (via Google Forms and distributed through social networks such as Facebook and Instagram) or on paper (in schools during regular school hours). Participation was voluntary, anonymous, and participants did not receive any compensation. The study was approved by the Ethics Committee of the Department of Psychology, University of Novi Sad (Approval Code: 202206142223_p3JQ). Informed consent was obtained from all participants.

### Measures

Serbian versions of questionnaires were used in the present study. All questionnaires were translated using a committee-based approach [40]. Three psychologists with PhD degrees (native Serbian speakers, fluent in English, experts in the fields of mental health and health behaviour change) independently translated the questionnaires, after which two additional psychologists reviewed the translations and provided their comments and suggestions on how to improve and refine the translations. Final versions of translations were achieved by consensus. The committee approach is a widely used procedure for questionnaire translation and it is considered a translation best practice in the process of cross-cultural adaptation of self-report measures [41].

**Vaccination Attitude Examination Scale** (VAX) [16] is a 12-item measure of general attitudes toward vaccinations. The VAX includes four subscales, with three items each: Mistrust of Vaccine Benefit (e.g., *I feel protected after getting vaccinated*), Worries about Unforeseen Future Effects (e.g., *Vaccines can cause unforeseen problems in children*), Concerns about Commercial Profiteering (e.g., *Vaccines make a lot of money for pharmaceutical companies, but do not do much for regular people*), and Preference for Natural Immunity (e.g., *Natural immunity lasts longer than a vaccination*). Each item is rated on a 6-point scale, from 1 (*strongly disagree*) to 6 (*strongly agree*).

**Vaccine Conspiracy Beliefs Scale** (VCBS) [42] consists of seven items to assess vaccine-specific conspiracy beliefs (e.g., *Pharmaceutical companies cover up the dangers of vaccines*). Respondents rated each item on a 7-point scale ranging from 1 (*strongly disagree*) to 7 (*strongly agree*). Internal consistency of the VCBS was high in the present sample (*α* = 0.95, *ω* = 0.95).

**Attitudes towards COVID-19 vaccination** were measured using three 7-point semantic differential items designed by Lueck & Spiers [43]. As the mass vaccination against COVID-19 in Serbia started approximately 18 months prior to recruiting the present sample, the assessment of attitudes toward COVID-19 vaccination was preceded by a short vignette. Participants were asked to imagine that the number of COVID-19 infections would increase over the next few months (with several thousand new cases and about a hundred deaths due to COVID-19 per day) and that health experts had unanimously agreed that adolescents aged 15 and older should receive the COVID-19 vaccine. After presenting the vignette, the stem, “My getting vaccinated against COVID-19 if the pandemic situation worsens would be …” was followed by the following pairs of items: *harmful-beneficial*, *unnecessary-necessary*, *bad-good*. In the present study, the internal consistency of the scale was high (*α* = 0.96, *ω* = 0.96).

**Intention to get vaccinated against COVID-19** was measured using the following three items: *I intend to get vaccinated against the coronavirus*; *I expect to get vaccinated against the coronavirus*; and *It is highly likely that I will get vaccinated against the coronavirus*, rated on a 7-point scale (from 1 = *completely untrue* to 7 = *completely true*). The assessment of the intention to get vaccinated against COVID-19 was also preceded by the vignette described above. The internal consistency of this measure was high (*α* = 0.98, *ω* = 0.98).

**Injections and Blood Draws subscale of the Medical Fear Survey – short version –** (MFS) [44] is a 4-item measure of fear of injections and blood draws. Participants were asked to rate how much fear or tension (0 = *no fear or concern at all*, 1 = *mild fear*, 2 = *considerable fear*, 3 = *intense fear*) they would experience in the listed situation (e.g., “*having blood drawn from your arm*”). Internal consistency reliability in the present sample was adequate (*α* = 0.83, *ω* = 0.83).

**Paranoia Worries Questionnaire** (PWQ) [45] is a 5-item measure of problematic worry focused on paranoid content (e.g., *Thinking about the possible attacks on me has made me feel stressed*). Items are rated on a 5-point scale (from 0 = *none of the time* to 4 = *all of the time*). Internal consistency reliability in the present sample was adequate (*α* = 0.85, *ω* = 0.86).

**Trust in healthcare** was assessed using three items: trust in doctors, the healthcare system, and the official medicine, rated on an 11-point scale (from 0 = *do not trust at all* to 10 = *trust completely*). Internal consistency of the scale was adequate in the present sample (*α* = 0.87, *ω* = 0.87).

### Data Analysis

Five competing models of the VAX structure were evaluated: (1) One-factor CFA model, with all 12 items loading on a single factor; (2) Four-factor oblique CFA model, with four correlated factors: Mistrust of Vaccine Benefit (items 1, 2, 3), Worries about Unforeseen Future Effects (items 4, 5, 6), Concerns about Commercial Profiteering (items 7, 8, 9), and Preference for Natural Immunity (items 10, 11, 12); (3) ESEM model (with target rotation where all cross-loadings are targeted to be as close to zero as possible), with four correlated factors and all cross-loadings freely estimated; (4) Bifactor-CFA model, with one general factor (G-factor), and four orthogonal specific factors (S-factors). In this model, items are allowed to load on the G-factor and onto their a priori S-factor; and (5) Bifactor-ESEM model (with orthogonal target rotation), with the same specifications as the bifactor-CFA model but all cross-loadings freely estimated. MLR (robust maximum likelihood) estimation method was used, as the VAX response scale includes six response options.

The following fit indices were used to evaluate the model fit: comparative fit index (CFI), Tucker-Lewis index (TLI), and root mean square error of approximation (RMSEA) with 90% confidence intervals (CI). CFI and TLI values above 0.95, and RMSEA 90% CI upper values below 0.05 were considered indicative of a good fit [46], whereas cut-offs of 0.90 and 0.10 for CFI/TLI and RMSEA 90% CI upper values, respectively, were considered acceptable [47]. The competing models were compared using the following recommendations by Chen [48] and Cheung and Rensvold [49]: decrement > 0.01 in CFI and TLI values, and increase > 0.015 in RMSEA values suggest that model provides a worse fit to the data compared to the previous model. Omega coefficient of composite reliability (*ω*) was computed to assess reliability, which has been shown to be appropriate for bifactor and ESEM solutions [31]. Typically, Omega above 0.70 is considered to indicate adequate reliability [50].

Evidence of convergent and discriminant validity of VAX scores was evaluated by examining associations with intention to get vaccinated against COVID-19, attitudes towards COVID-19 vaccination, trust in healthcare, vaccine conspiracy beliefs, medical fears, and paranoid worry scores, using structural equation modeling (SEM). VAX latent factors from the final solution were used as predictors, whereas the latent factors of the abovementioned measures were used as criteria [51]. Separate SEMs were run for each criterion.

Missing data were handled using the Full Information Maximum Likelihood (FIML) procedure^1^. Cases (*n* = 3) with missing values on all VAX items were deleted prior to testing the factor structure of the VAX, and cases with missing values on all items on a measure used to investigate evidence on convergent and discriminant validity were deleted prior to examining associations with that measure. The positively worded items of the VAX (items 1, 2, and 3) were recoded prior to analyses to indicate negative attitudes towards vaccination. The data analyses were performed in Mplus 8.7 [53] and JASP 0.11.1.0 [54].

## Results

### The competing measurement models of vaccination attitudes

Table 1 shows the goodness-of-fit indices of the competing models of the VAX structure, whereas the parameter estimates are presented in Table 2. The single-factor CFA model provided a poor fit to the data, whereas the four-factor CFA and the bifactor-CFA achieved an acceptable fit to the data. The ESEM model showed a good fit to the data based on the CFI values and an acceptable fit based on the TLI and RMSEA values. The bifactor-ESEM model achieved a good fit to the data and demonstrated superiority to the competing models, as indicated by differences in CFI, TLI, and RMSEA values. However, the choice of the most appropriate model should not be guided solely by the goodness-of-fit indices [31], so we proceeded with evaluating parameter estimates obtained from the competing models.

**Table 1:** Goodness-of-fit statistics of the competing measurement models

Model	χ^2^	df	CFI	TLI	RMSEA (90% CI)
Single-factor CFA	1957.38	54	0.556	0.458	0.210 (0.202, 0.218)
Four-factor CFA	345.70	48	0.931	0.905	0.088 (0.079, 0.097)
ESEM	146.84	24	0.971	0.921	0.080 (0.068, 0.093)
Bifactor-CFA	272.18	42	0.946	0.916	0.083 (0.074, 0.092)
Bifactor-ESEM	37.54	16	0.995	0.979	0.041 (0.024, 0.058)

*Note*: CFA = confirmatory factor analysis, ESEM = exploratory structural equation modeling, χ^2^ = chi square, df = degress of freedom, CFI = comparative fit index, TLI = Tucker-Lewis Index, RMSEA = root mean square error of approximation, CI = confidence interval

#### CFA versus ESEM

Based on the recommendations in the literature [32], we first compared parameter estimates from the first-order four-factor CFA and ESEM models. The inspection of parameter estimates showed that factor loadings and Omega coefficients were generally high in both four-factor CFA (*λ* range from 0.580 to 0.951; *M*_*λ*_ = 0.803; *ω* = 0.796 to 0.924) and ESEM (*λ* range from 0.539 to 0.989; *M*_*λ*_ = 0.769; *ω* = 0.794 to 0.928) models. All S-factors in the ESEM solution were well-defined, with the *Mistrust of Vaccine Benefit* factor defined best. Factor correlations (see Additional File 2) were lower in the ESEM (range from 0.179 to 0.710; *M*_*r*_ = 0.496) compared to the four-factor CFA model (range from 0.328 to 0.759: *M*_*r*_ = 0.570), indicating that the factors were more clearly differentiated in the ESEM solution. The inspection of cross-loadings in the ESEM model showed that the majority of cross-loadings were low (for example, out of 36 cross-loadings, only 11 were above |0.10|, but none was greater than |0.20|) and statistically non-significant (out of 36 cross-loadings, only seven were statistically significant), thus not interfering with the interpretation of the S-factors. These results, along with the better fit of the ESEM compared to the four-factor CFA model, suggest that ESEM model is a better representation of the data and should be contrasted with the bifactor-ESEM model.

**Table 2:** Parameter estimates (standardized factor loadings and omega coefficients) from the competing measurement models

	CFA	ESEM				Bifactor-CFA		Bifactor-ESEM				
	FO	Mistrust	Worries	Concerns	Preference	G	S	G	Mistrust	Worries	Concerns	Preference
Mistrust												
VAX1	.880**	.884**	.118**	-0.078	.054*	.579**	.654**	.537**	.686**	0.012	-0.007	0.009
VAX2	.854**	.792**	− .140**	.186**	-0.044	.527**	.662**	.524**	.670**	− .189**	-0.084	− .087**
VAX3	.951**	.989**	0.033	-0.085	0.018	.546**	.798**	.477**	.851**	-0.028	.085**	0.026
*ω*	*0.924*	*0.928*					*0.884*		*0.906*			
Worries												
VAX4	.580**	-0.128	.738**	-0.075	-0.018	.301**	.563**	.305**	− .193**	.602**	.197**	0.065
VAX5	.808**	0.054	.743**	0.039	0.043	.546**	.651**	.585**	− .061*	.552**	-0.048	0.017
VAX6	.848**	.137**	.656**	0.18	-0.009	.657**	.486**	.695**	0.015	.485**	-0.087	-0.045
*ω*	*0.796*		*0.794*				*0.706*			*0.713*		
Concerns												
VAX7	.735**	0.027	0.187	.581**	0.003	.723**	0.257	.688**	0.042	.128**	.292**	-0.003
VAX8	.751**	-0.098	0.127	.657**	0.089	.746**	0.213	.725**	− .054*	.086*	.268**	0.054
VAX9	.837**	0.08	− .121**	.875**	0.036	.867**	-0.121	.847**	.145**	− .106**	**0.093**	-0.008
*ω*	*0.819*			*0.8*			*0.251*				*0.288*	
Preference												
VAX10	.720**	0.022	.119*	0.124	.539**	.616**	.352**	.584**	0.006	.116**	.199**	.396**
VAX11	.816**	-0.053	-0.045	-0.061	.942**	.563**	.653**	.588**	− .075**	-0.003	0.018	.577**
VAX12	.858**	0.062	-0.041	0.022	.828**	.643**	.551**	.678**	0.018	-0.03	− .135**	.566**
*ω*	*0.841*				*0.839*	*0.933*	*0.7*	*0.935*				*0.711*

*Note*: FO = first-order, VAX1-VAX12 = VAX items, *ω =* Omega coefficient of reliability, G = general factor, S = specific factor, Mistrust = Mistrust of Vaccine Benefit (VAX), Worries = Worries about Unforeseen Future Effects (VAX), Concerns = Concerns about Commercial Profiteering (VAX), Preference = Preference for Natural Immunity (VAX).

Target factor loadings are marked in bold. Omega coefficients are italicized

#### ESEM versus Bifactor-ESEM

The G-factor of vaccination attitudes obtained in the bifactor-ESEM solution was well-defined (*λ* range from 0.305 to 0.847; *M*_*λ*_ = 0.603; *ω* = 0.935). Three S-factors were well-defined by target indicators and had Omega > 0.70: *Mistrust of Vaccine Benefit*, *Worries about Unforeseen Future Effects*, and *Preference for Natural Immunity* (see Table 2). The magnitudes of factor loadings were generally comparable for G-factor and these three S-factors, but it is noticeable that items 1, 2, and 3 loaded more strongly on their target S-factor (*Mistrust of Vaccine Benefit*) than on the G-factor. However, one S-factor (*Concerns about Commercial Profiteering*) was poorly defined (*λ*s < 0.30; *ω* = 0.288) and its items had the strongest loadings on the G-factor. Given the superiority of the bifactor-ESEM model as the representation of the VAX data, this solution was retained for the subsequent validity analyses.

### Association between vaccination attitudes and convergent and discriminant measures

Using separate SEM analyses for each criterion (see Table 3 for parameter estimates, and see Additional File 3 for model fit indices), we regressed intention to get vaccinated against COVID-19, attitudes towards COVID-19 vaccination, trust in healthcare, vaccine conspiracy beliefs, medical fears, and paranoid worry latent factors on the four specific vaccination attitude factors and the G-factor obtained using the bifactor-ESEM. The results showed that the G-factor of vaccine attitudes was the strongest predictor of and was closely associated with vaccine conspiracy beliefs (*β* = 0.767, *p* < .01), attitudes towards COVID-19 vaccination (*β* = − 0.575, *p* < .01), and intention to get vaccinated against COVID-19 (*β* = − 0.536, *p* < .01). The G-factor and S-factor *Mistrust of Vaccine Benefit* had similar predictive values in predicting trust in healthcare (*β* = − 0.363, *p* < .01; *β* = − 0.369, *p* < .01, respectively). Among the S-factors, only *Mistrust of Vaccine Benefit* had robust and consistent associations with the convergent measures, whereas the contribution of the remaining three S-factors was generally weak and nonsignificant^2^ (see Table 3). Discriminant validity of the VAX scores was supported by weak positive associations of the G-factor with medical fears (*β* = 0.135, *p* < .01) and paranoid worry (*β* = 0.238, *p* < .01). None of the three S-factors which showed evidence of validity and specificity in the bifactor-ESEM solution was a significant predictor of medical fears and paranoid worry.

**Table 3:** Structural regressions of convergent and discriminant measures on bifactor-ESEM VAX factors

	Intention to get vaccinated against COVID-19	Attitudes towards COVID-19 vaccination	Trust in healthcare	Vaccine conspiracy beliefs	Medical fears	Paranoid worry
	*β* (SE)	*β* (SE)	*β* (SE)	*β* (SE)	*β* (SE)	*β* (SE)
G-factor	− 0.536** (0.038)	− 0.575** (0.049)	− 0.363** (0.045)	0.767** (0.032)	0.135** (0.046)	0.238** (0.043)
S-factors						
Mistrust of Vaccine Benefit	− 0.392** (0.047)	− 0.426** (0.056)	− 0.369** (0.043)	0.220** (0.037)	− 0.015 (0.042)	0.002 (0.043)
Worries about Unforeseen Future Effects	0.037 (0.040)	0.034 (0.064)	0.061 (0.050)	0.097** (0.036)	− 0.005 (0.053)	− 0.079 (0.053)
Concerns about Commercial Profiteering	0.130** (0.040)	0.061 (0.124)	− 0.035 (0.080)	0.096 (0.130)	− 0.099 (0.061)	− 0.183** (0.062)
Preference for Natural Immunity	0.080 (0.041)	0.033 (0.054)	0.101* (0.045)	0.087* (0.037)	− 0.098 (0.056)	− 0.034 (0.055)
R^2^	0.466**	0.518**	0.283**	0.658**	0.038	0.098**

*Note*: *β* = standardized regression coefficient, SE = standard error, G-factor = general factor, S-factor = specific factor. R^2^ = percentage of explained variance

** *p* < .01, * *p* < .05

## Discussion

The present study aimed to evaluate competing models of the factor structure of the VAX scale, a widely used tool designed to assess attitudes toward vaccination. To our knowledge, this is the first study to investigate the structural, convergent, and discriminant validity of the VAX scale among youths using the bifactor and ESEM approaches.

The results of the present study suggest that the representation of the VAX scale’s factor structure might be refined and improved by applying the bifactor-ESEM approach. We found that the bifactor-ESEM model provided the best fit to the data, and that the representation of the VAX structure benefits from incorporating the cross-loadings, which allows each item to reflect both specific domains of vaccination attitudes and a global component. The results indicated that three S-factors (*Mistrust of Vaccine Benefit, Worries about Unforeseen Future Effects*, and *Preference for Natural Immunity*) tapped into specific vaccination attitudes, and the S-factor *Concerns about Commercial Profiteering* retained a small amount of specificity once the G-factor was taken into account. The latter finding calls into question the specific value of items covering concerns about “profit over people,” and suggests that positing *Concerns about Commercial Profiteering* as a specific domain of vaccination attitudes among youth is problematic. Low level of specificity of this S-factor observed in the present sample suggests that retaining this factor in subsequent analyses might be questionable and indicates that researchers should carefully investigate whether it is meaningful to drop it from the further analyses (for example, when examining the predictive validity of the VAX scores). However, three items of this subscale loaded strongly on the G-factor, so they can be considered useful constituents of general vaccination attitudes. Our findings support the notion that understanding the representation of multidimensional questionnaires can be improved by applying the bifactor-ESEM approach, which has proved to be useful in understanding the structure of scales aimed at measuring domains of well-being [55, 56], motivation [57, 58], and social support [59], to name a few.

The results of convergent and discriminant validity further supported the value of using the bifactor-ESEM approach. We found that the G-factor of vaccine attitudes was a strong and robust predictor of scores on convergent measures assessing vaccine conspiracy beliefs, attitudes towards COVID-19 vaccination, intention to get vaccinated against COVID-19, and trust in healthcare. Among the S-factors, only *Mistrust of Vaccine Benefit* had a unique predictive value for all four convergent measures after partialling out the effects of the G-factor. The remaining S-factors demonstrated little validity in predicting scores on the convergent measures. These findings suggest that, beyond a general negative stance towards vaccination, belief in the safety and efficacy of vaccines (i.e., trust/mistrust of vaccine benefit) might be the most important facet of vaccination attitudes in understanding vaccine-related behaviors. These results are in line with previous studies indicating that general attitudes towards COVID-19 vaccines, as assessed by a semantic differential scale, are the strongest predictor of COVID-19 vaccination intentions [60, 61], and are closely associated with trust in science and COVID-19 conspiracy beliefs [61]. Furthermore, previous studies also found that distrust of the vaccine’s safety was the most important determinant of COVID-19 vaccine hesitancy [62]. It is important to note that previous studies using the scores on four VAX subscales to investigate the associations with vaccination hesitancy typically found that all four negative attitude domains were significantly associated with measures of vaccine hesitancy [5, 63, 64], and that scores on the *Mistrust of Vaccine Benefit* subscale were most closely associated with vaccine hesitancy [65, 66]. However, the results of the present study suggest that the associations between different domains of vaccination attitudes and external criteria are driven primarily by general attitudes towards vaccination and not specific components, except in the case of mistrust of vaccine benefit. These findings warrant further investigation and should be replicated using samples from different countries and age groups.

Finally, weak positive associations of the G-factor with medical fears and paranoid worry, and nonsignificant associations of these two measures with scores on S-factors which showed evidence of validity and specificity over and above the G-factor indicated evidence of the discriminant validity of the VAX scores obtained in the bifactor-ESEM solution.

### Limitations and future directions

This study is not without limitations. First, we recruited a convenience sample of adolescents and young adults, which limits the generalizability of results. Our findings should be replicated on a representative sample of adolescents and across other age groups. Second, we sampled participants from a single country, which limits the cross-cultural generalizability of our findings. The meaning of vaccines and vaccinations is rooted in socio-cultural context [67], and vaccination attitudes vary considerably across cultures [68]. Thus, future studies should investigate the structure of vaccination attitudes as assessed by the VAX across different cultural contexts to enable investigation of the scale’s cross-cultural measurement invariance. Third, we used two convergent measures (intention to get vaccinated and attitudes towards vaccination) related to COVID-19 vaccines, so future studies should investigate whether our findings hold up when measures are used that refer to other specific vaccines, such as influenza, HPV, or HIV. Some studies show that attitudes towards different vaccines may vary [69], and that newer vaccines are typically met with greater skepticism and hesitancy [70]. Thus, the predictive value of general and specific vaccination attitudes could be different in predicting behaviors concerning different specific vaccines. Fourth, we relied on self-report measures, which is an important limitation of the present study as it may increase common method variance bias [71]. Future studies should use methods other than self-report; for example, the predictive validity of vaccination attitudes as assessed by the VAX should be investigated in relation to objective measures (e.g., objective data on vaccination status) or other-reports (e.g., parent report) of vaccine-related behaviors. Finally, we also suggest that researchers in future studies present the VAX items in a randomized order to further minimize common method bias, since items from the same subscale are placed one after another in the original English version of the VAX.

## Conclusions

The present study highlights the importance of making a distinction between general and specific components of vaccination attitudes as assessed by the VAX scale in order to avoid erroneous conclusions about the structure of this scale. Taking into account the G-factor of vaccination attitudes as assessed by the VAX scale appears to provide a clearer and more refined picture than a simple four-factor solution, and thus models that aim to identify sources of construct-relevant multidimensionality should be evaluated prior to performing subsequent analyses using the scores obtained on this scale. Our findings have some important implications for scoring and applying the VAX scale in research and applied settings. We argue that bifactor-ESEM model should be tested when using the VAX in order to provide a more precise and complete picture of vaccination attitudes structure and to disaggregate the variance attributed to the general and specific factors of attitudes toward vaccination. Our results suggest that researchers should obtain both the global and specific components of vaccination attitudes when using the VAX, and that relying solely on one VAX factor or four VAX factors obtained using the CFA may be inappropriate, and consequently, may generate potentially misleading practical recommendations. Finally, we argue that researchers should use VAX factor scores from preliminary measurement model instead of simply averaged or summed subscale scores in the convergent, discriminant, and predictive validity analyses.

### Electronic supplementary material

Below is the link to the electronic supplementary material.

Supplementary Material 1

Supplementary Material 2

Supplementary Material 3

Supplementary Material 4

## Data Availability

All materials, including the Serbian translation of the VAX scale, datasets, and analysis syntaxes can be found at: https://osf.io/3b4fy/.

## Footnotes

1 We also applied multiple imputation to handle missing data and compared the findings to those obtained using the FIML. The results were highly similar indicating that the findings are robust to the presence of missing data [52].

2 In the Additional File 4 we report the results of convergent and discriminant validity analyses obtained using the commonly used four-factor CFA model of the VAX. These results indicate substantial discrepancies in the relations between *Concerns about Commercial Profiteering* and convergent measures when bifactor-ESEM and standard four-factor CFA solutions are used, supporting the benefits of applying the bifactor-ESEM model.
