# Supplementary material for: Vaccination Attitudes Examination (VAX) Scale: a Bifactor-ESEM approach in a youth sample (15–24 years)
Source: BMC Psychol. 2023 Oct 23;11:351. doi: 10.1186/s40359-023-01388-9 (PMC10594745; doi:10.1186/s40359-023-01388-9)
Supplement: Supplementary file 1 — Supplementary Material 1 [file 40359_2023_1388_MOESM1_ESM.docx]

**Additional File 1**

*Sample Description*

|  | *n (%)* |
| --- | --- |
| Gender |  |
| Female | 475 (39.5%) |
| Male | 317 (59.2%) |
| Did not answer | 11 (1.3%) |
| Education level of mother |  |
| Elementary school or less | 22 (2.7%) |
| Secondary school | 377 (47%) |
| University | 396 (49.3%) |
| Did not answer | 11 (1%) |
| Education level of father |  |
| Elementary school or less | 27 (3.4%) |
| Secondary school | 451 (56.2%) |
| University | 314 (39.1%) |
| Did not answer | 11 (1.3%) |
| Self-rated family’s financial situation |  |
| Poor | 32 (4%) |
| Below average | 77 (9.6%) |
| Average | 257 (32%) |
| Good | 261 (32.5%) |
| Excellent | 141 (17.5%) |
| Did not answer | 35 (4.4%) |
| Vaccination Status |  |
| Vaccinated | 197 (24.5%) |
| Unvaccinated | 581 (72.4%) |
| Did not answer | 25 (3.1%) |
| Personal history of COVID-19 |  |
| Yes | 317 (39.5%) |
| No | 410 (51%) |
| Did not answer | 76 (9.5%) |
