# Supplementary material for: Vaccination Attitudes Examination (VAX) Scale: a Bifactor-ESEM approach in a youth sample (15–24 years)
Source: BMC Psychol. 2023 Oct 23;11:351. doi: 10.1186/s40359-023-01388-9 (PMC10594745; doi:10.1186/s40359-023-01388-9)
Supplement: Supplementary file 2 — Supplementary Material 2 [file 40359_2023_1388_MOESM2_ESM.docx]

**Additional File 2**

*Factor Correlations in the ESEM (Above the Diagonal) and the Four-Factor CFA (Below the Diagonal) Models*

| Factor | 1 | 2 | 3 | 4 |
| --- | --- | --- | --- | --- |
| 1. Mistrust of Vaccine Benefit | - | .179 | .602 | .374 |
| 2. Worries about Unforeseen Future Effects | .328 | - | .603 | .509 |
| 3. Concerns about Commercial Profiteering | .610 | .732 | - | .710 |
| 4. Preference for Natural Immunity | .404 | .584 | .759 | - |

*Note:* All correlations are significant at *p* < .01.
