# Supplementary material for: Vaccination Attitudes Examination (VAX) Scale: a Bifactor-ESEM approach in a youth sample (15–24 years)
Source: BMC Psychol. 2023 Oct 23;11:351. doi: 10.1186/s40359-023-01388-9 (PMC10594745; doi:10.1186/s40359-023-01388-9)
Supplement: Supplementary file 3 — Supplementary Material 3 [file 40359_2023_1388_MOESM3_ESM.docx]

**Additional File 3**

*Model Fit for the SEM Models Testing Associations between Vaccination Attitudes (Bifactor-ESEM Solution) and Convergent and Discriminant Measures*

|  | χ^2^ _(df)_ | CFI | RMSEA (90% CI) |
| --- | --- | --- | --- |
| Intention to get vaccinated against COVID-19 | 81.40 _(47)_ | .995 | .030 (.019, .041) |
| Atttitudes towards COVID-19 vaccination | 94.46 _(47)_ | .993 | .036 (.025, .046) |
| Trust in healthcare | 112.36 _(47)_ | .988 | .042 (.032, .052) |
| Vaccine conspiracy beliefs | 332.14 _(109)_ | .974 | .051 (.045, .057) |
| Medical fears | 116.64 _(61)_ | .990 | .034 (.024, .043) |
| Paranoid worry | 216.26 _(76)_ | .976 | .048 (.041, .056) |

*Note*: χ^2^ = chi square, df = degress of freedom, CFI = comparative fit index, RMSEA = root mean square error of approximation, CI = confidence interval.
