# Supplementary material for: Vaccination Attitudes Examination (VAX) Scale: a Bifactor-ESEM approach in a youth sample (15–24 years)
Source: BMC Psychol. 2023 Oct 23;11:351. doi: 10.1186/s40359-023-01388-9 (PMC10594745; doi:10.1186/s40359-023-01388-9)
Supplement: Supplementary file 4 — Supplementary Material 4 [file 40359_2023_1388_MOESM4_ESM.docx]

**Additional File 4**

*Structural Regressions of Convergent and Discriminant Measures on VAX Factors Obtained in the Four-Factor CFA Solution*

|  | Intention to get vaccinated against COVID-19 | Attitudes towards COVID-19 vaccination | Trust in healthcare | Vaccine conspiracy beliefs | Medical fears | Paranoid worry |
| --- | --- | --- | --- | --- | --- | --- |
|  | *β* (SE) | *β* (SE) | *β* (SE) | *β* (SE) | *β* (SE) | *β* (SE) |
| Mistrust of Vaccine Benefit | -.552** (.041) | -.496** (.048) | -.428** (.057) | .231** (.049) | .006 (.063) | .043 (.060) |
| Worries about Unforeseen Future Effects | -.104 (.055) | -.003 (.056) | -.036 (.074) | .147** (.052) | .070 (.076) | .018 (.075) |
| Concerns about Commercial Profiteering | -.075 (.089) | -.326** (.091) | -.265* (.122) | .445** (.084) | .103 (.133) | .088 (.128) |
| Preference for Natural Immunity | -.015 (.059) | -.040 (.056) | .149* (.071) | .125* (.055) | -.077 (.087) | .070 (.085) |
| R^2^ | .430** | .517** | .287** | .662** | .014 | .036* |
| χ^2^ _(df)_ | 431.21_(80)_ | 421.08_(80)_ | 430.13_(80)_ | 628.20_(142)_ | 447.17 _(94)_ | 543.47 _(109)_ |
| CFI | .952 | .950 | .936 | .944 | .936 | .927 |
| RMSEA (90% CI) | .074 (.068, .081) | .074 (.067, .081) | .075 (.068, .082) | .066 (.060, .071) | .069 (.062, .075) | .071 (.065, .077) |

*Note*: *β* = standardized regression coefficient, SE = standard error, R^2^ = percentage of explained variance, χ^2^ = chi square, df = degress of freedom, CFI = comparative fit index, RMSEA = root mean square error of approximation, CI = confidence interval.

***p* < .01, **p* < .05
